# Address Delivered before the Pennsylvania Society of Dental Surgeons

**Published:** 1852-01

**Authors:** 


*Address delivered before the Pennsylvania Society of Dental Surgeons,* at Sansom Street Hall, Philadelphia, October 10th, 1851. By Elisha Townsend, D. D. S., etc. etc., pp. 40.

We cannot give a better idea of the general scope of the above address, than by quoting a few passages from it. After stating the objects of the society, before which it was delivered, the author says:

"Individual improvement in the knowledge of our profession, and the advancement of the profession itself, thus recognised and provided for by our association, are alike obligatory upon us, whether we would acquit ourselves worthily towards our patients in private practice, or discharge the implied obligations which we owe to the community and to the world: and happily, these aims, and the means of accomplishing them are in all particulars, either identical or mutually helpful. The lower and narrower interests of individuals and communities may clash with each other, but the higher and nobler, grow more liberal, consistent and harmonious as they rise in dignity and value. It is the appetites that squabble over their prey; the moral sentiments ever co-operate and harmonize in all their movements. This principle is so true in all possible applications, that we may safely carry it with us for criticism and direction throughout the whole region of speculation and practice.

"The methods or agencies of this improvement of the individual, including both practitioner and pupil, and the necessary advancement of the science which they are bound to carry forward with them, I propose now to consider; and for this purpose I suggest, that the questions of Dental Societies, Dental Colleges, and the reactive effect of that public opinion which they have the power to create, afford the best division and array of the points to be discussed. These are subjects of special interest at the present juncture of our profession's misfortunes; and our opinions and actions upon them are of great moment to its welfare.

"But a little while ago, the student of dentistry caught up the scattered hints of theory, and occasional directions in practice, then accessible, as he best could. It was a mere matter of good fortune to meet with the right method and man in his need, and his attainments in the art depended so much upon his own discoveries, and so little upon his tuition proper, that the idea of self-made dentists and self dependence in the pursuit of improvement very naturally got a strong hold in the minds of some of our best men, and still keeps its place there with all the tenacity of a prejudice based upon experience. Self-dependence and self-development are fundamental truths indeed, but the notions of them, gained when they were the sole reliance for attainment, or nearly so, must not be carried forward into the changed circumstances to which they no longer apply.

"The stout hearts and good heads of that day, made noble achievements in the field of discovery and reform, and acquired much that now asks better *methods* of teaching, than sufficed before the business of dentistry had grown into a collection of scientific facts and principles, rich and full, for its own uses, as any other branch of the healing art.

"Professions, like individuals, walk and run by the same self-moving forces with which they crept, but very differently exerted and arrayed, and in a very different attitude to objects, guides and helps—I mean here to say, that we stand in the present, with our faces towards the future; and as experience is not the measure of our hopes, so its formulas must not limit or prescribe our aims and methods, nor must its authority be allowed to control or curb our liberties. When men talk of experience as the test of truth, they should be careful not to confine the thought to *past* experience *wholly;* there is a *future* experience, whose office it is to try the ideas of to-day, and it is its office, too, to enlarge the partial truth of yesterday, into wider views, and to correct the old fact by that fuller truth which is to serve the world's turn to-morrow."

On the subject of association, Dr. Townsend remarks:

"The principle of association holds as well in the cultivation of professional learning, as in any other matter of human action, which can be helped by mutuality of communication, and reciprocity of service. The objects of the great endeavor have such variety of character, and each has such diversity of aspects and relations, as require the greatest variety of minds and conditions in the observers for their thorough investigation; especially, during the earlier periods of development, while new facts are in demand and new views of old facts are required, for the adjustment and harmony of the whole; most especially, while a science is just rounding into form, does it demand, not only the services, but the united services of every grade of attainment and every order of mind that exists among its cultivators. Of a hundred men, ranking on the same general level of reputation and real worth, who may conduct their investigations separately, no two shall see any one thing in all particulars, alike, or consider any one subject, under exactly the same aspects, though each shall observe and consider it justly, in the partial view he takes of it. Whoever has looked into this matter, knows, that theories, investigations and processes, both mental and manual, conducted by the best minds alone, are almost sure to include elements not essential, and to overlook or miscalulate others most material to the issue—faults which far inferior minds, that happen to be free from disturbing prepossessions, can easily correct. And when we add to all, the difference of original capacity and individual influences, the equally great difference of opportunity, which the varied condition of a great number of men actively engaged in professional practice affords, the importance of free communication and mutual correction, to accuracy and completeness of knowledge, appears in a most imposing light. As to the means of this indispensable interchange of thought and discovery, after allowing their due importance and place to books and periodicals, other and most important uses are found to belong appropriately to the method by personal intercourse in deliberative and polemical assemblies of adepts. However adequate the press might appear, at first sight, to supply all this need, the popular measures for the exchange of knowledge which are now in general use, are in proof of the superior availability of oral discussion in certain respects of very high value. The gravest, most recondite, and the oldest scientifie societies, are now employing conventions and stated assemblies for the cultivation of their respective departments of study, and the early dissemination of discovery.

"There is something in the necessities of professional movements which it is felt associated action can best answer, or otherwise, the libraries accumulating through centuries, and the periodicals numerous and able as can be wished, and the first class daily papers, served by phonography and the telegraph, would fully answer the need, and spare the expense of toil and travel which is everywhere encountered in this method of prosecuting scientific improvement. Without attempting a complete list, or even intending a formal array, I need but refer for instances, to such bodies as the American Society of Physicians and Surgeons, the American Association for the advancement of Science, the Academy of Natural Sciences of Philadelphia, the Boston Society of Natural History, the American Association for the advancement of Education, the College of Teachers, embracing the active educators of the great Mississippi Valley, the American Society of Dental Surgeons, and such local and state societies as our own. These, a few of many, show that the cultivators of the sciences, the practitioners of the learned professions, who feel the necessity of a forward movement, and the friends of general education, are all using the method of personal conference, discussion and oral communication, on some common and clear perception of its peculiar advantages, and of its remunerating value in the service of general learning. Even in the membership of the few societies here named, might be found, perhaps, the names of all the men in our country known to fame, and deserving it by valuable services to the highest interests of the world; especially noteworthy is it, that the men who are the accredited authorities of the day, are found most punctual and active in these parliaments of progress. In the morning newspapers we meet the names, and brief exhibits of the last best ideas, of the very men whose works are the gems of our libraries. Naturalists, the medical faculty in all its specialities, the educators in general literature, from the common school to the oldest colleges; teachers of the deaf and dumb, and superintendants of insane asylums; with I know not how many more divisions of the great army of occupation and advance, are *our* warranty for the necessity and utility of such voluntary associations as ours, so far as the highest example can assure opinion, and support the resulting practice. I might safely rest my point here, and let the demonstration, afforded by universal assent, make its appeal to our brethren in the dental profession, for their active co-operation in this mode of individual and mutual improvement, and I do so rest it, none the less that I also appeal to the great *principle* of association, which underlies all experience of its excellent policy, in order that any imagined difference of conditions and interests belonging to any particular profession may be met and obviated. So far as the argument respects our own profession, I think I should be specially warranted in turning short upon opposition and indifference, with the answer, that it is not for men whose elementary studies and every day observation teaches them the sympathies that link the various organs and functions of the human body into unity and harmony, it is not for them to dispute, or to doubt, or neglect, the dependence of every difference of endowment, and the mutuality of all diversity of office, which relates to one system, and ministers to one end. This admirable contexture of multiform organisms, which we call our body, and by which we are related to the universe, and through which we govern the material world, is indeed the best, if not the only perfect type of a church fraternity, or circle society of men. The great apostle of the Gentiles recognises this truth, and makes his own characteristic use of it, in this nervous language, 'we are many, yet but one body. If one member suffer, all the members suffer with it, or if one member be honored, all the members rejoice with it.' The anatomical and physiological facts in the human constitution, to which this bold figure of speech refers, are familiar to the men whom I now address, and it only asks a moment's reflection to feel their application to groups and aggregates of men, as clear as they are in the individual man, whence their force is borrowed."

If space permitted, we would copy several other pages from this able and beautifully written address; indeed, we would be glad to publish it entire, feeling assured that its perusal would give our readers as much pleasure as we derived from reading it, but we have already quoted so much that we have not room for the comments which we had designed making. A very good idea, however, of the general scope of the address, may be formed from the passages which we have quoted, and of the ability displayed by the author, in the management of his subjects.

